# Different exercises can modulate the differentiation/maturation of neural stem/progenitor cells after photochemically induced focal cerebral infarction

**DOI:** 10.1002/brb3.1535

**Published:** 2020-01-27

**Authors:** Saho Morishita, Kazuya Hokamura, Akira Yoshikawa, Nobuhide Agata, Yoshihiro Tsutsui, Kazuo Umemura, Tatsuro Kumada

**Affiliations:** ^1^ Department of Health and Nutritional Sciences Faculty of Health Promotional Sciences Tokoha University Hamamatsu Japan; ^2^ Department of Pharmacology Hamamatsu University School of Medicine Hamamatsu Japan; ^3^ Department of Physiology Showa University School of Medicine Tokyo Japan; ^4^ Faculty of Health and Medical Sciences Tokoha University Hamamatsu Japan

**Keywords:** cerebral infarction, differentiation/maturation, exercise, neurogenesis

## Abstract

**Introduction:**

Exercise therapies during rehabilitation significantly promote recovery from various deficits after cerebral infarction, which is mediated by neuronal plasticity with distinct inputs. Although adult neurogenesis can also be modulated by neuronal activity before synaptogenesis, how distinct exercises contribute to the neurological reorganization of the injured cerebral cortex remains unclear. In the present study, we aimed to elucidate the effects of different exercise therapies on motor recovery and neuronal reorganization after photochemically induced focal cerebral infarction.

**Methods:**

Here, we examined the effects of three different exercises—(a) forced lower‐intensity and (b) higher‐intensity treadmill exercises, and (c) voluntary exercise with wheel running—on motor recovery and adult neurogenesis in a rat model of focal cerebral infarction. Photochemically induced thrombosis (PIT) was used to generate focal infarction in rats that was mostly confined to their motor cortices.

**Results:**

Beam walking tests showed that recovery after PIT‐induced cortical infarction differed in acute and chronic stages and was influenced by the type of exercise. Furthermore, forced low‐intensity training had more positive effects on functional recovery than other exercises or control. To evaluate the production of newly generated cells including *de novo* neurogenesis, we performed lineage analysis with BrdU labeling and immunofluorescence experiments. Lower‐intensity treadmill exercise increased the number of BrdU/NeuN colabeled cells, but not total BrdU‐retaining or BrdU/Sox2‐colabeled cells, in the peri‐infarct region of the ipsilateral cortex. In contrast, high‐intensity treadmill or voluntary exercises had the opposite effects.

**Conclusions:**

These results suggest that neuronal maturation can be differently modulated by distinct exercises and that low‐intensity treadmill exercise could result in more potent generation of mature neurons. This also suggests the possibility that the generation of neural stem/progenitor cells and differentiation might be modulated by rehabilitation‐mediated neural plasticity.

## Graphical abstract

Lower‐intensity treadmill exercise could increase the number of BrdU/NeuN colabeled cells, but not total BrdU‐retaining and BrdU/Sox2‐colabeled cells, in the peri‐infarct region of the ipsilateral cortex. Interestingly, high‐intensity treadmill or voluntary exercises exerted the opposite effects.

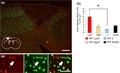

## 1 INTRODUCTION

During the rehabilitation period after a stroke, physical exercise plays a significant role in recovery from various deficits including motor dysfunction, which is mediated by neuronal plasticity (Nudo, 2013). Different exercises are believed to be accompanied by distinct activities of sensory motor circuits to affect neuronal plasticity. Furthermore, there is accumulating evidence that different types of physical exercises such as forced and voluntary running can affect recovery from motor or memory dysfunction (Choi et al., 2018; Cotman, Berchtold, & Christie, 2007; So et al., 2017).

Adult neurogenesis might be involved in reorganization of the injured brain. Thus, it is important to understand how physical exercise can lead to neurogenesis in the injured brain (Cotman, 2002; Cotman et al., 2007; Vivar & van Praag, 2017). It is widely believed that neuronal plasticity is prominent in the developing brain, but with increasing age becomes mostly lost and limited to only restricted events such as synaptogenesis. However, recent reports suggest that these abilities could remain dormant in adults and that adult neurogenesis could be also regulated by neuronal activity (Hubener & Bonhoeffer, 2014; Karadottir & Kuo, 2018; Snyder, 2019), which might be required for recalibration.

Central nervous system (CNS) injury often induces the generation of different types of cells derived from resident neural stem cells, which can reduce tissue damage and facilitate functional recovery (Sabelstrom et al., 2013). Although neurogenesis only occurs in restricted regions called neurogenic niches, namely the subventricular zone (SVZ) and subgranular zone of the intact adult forebrain, previous reports suggest that ischemic injury induces further adult neurogenesis not only in the SVZ but also in other regions such as those located in layer I of the cerebral cortex (Arvidsson, Collin, Kirik, Kokaia, & Lindvall, 2002; Faiz et al., 2015; Feliciano & Bordey, 2013; Komitova, Mattsson, Johansson, & Eriksson, 2005; Ohira et al., 2010; Ziemka‐Nalecz & Zalewska, 2012). The restarting of adult neurogenesis thus has the potential to trigger and stimulate regeneration following brain injury (Marlier, Verteneuil, Vandenbosch, & Malgrange, 2015; Zhang, Zhang, & Chopp, 2016). Therefore, it is important to determine whether there are conditions that favor the production of new neural stem cell‐derived cells that are linked to motor recovery.

Accordingly, how distinct exercises differentially contribute to the neurological reorganization of the brain after cerebral infarction remains unknown. To address this, in the current study, we examined the effects of different exercise programs (forced exercise by treadmill running with lower‐ and higher‐intensity and voluntary exercise with wheel running) on motor recovery after focal cerebral infarction caused by photochemically induced thrombosis (PIT). We also assessed whether these exercises could affect the distribution of newly generated cells 4 weeks after injury.

## 2 MATERIALS AND METHODS

### 2.1 Induction of focal cerebral stroke by PIT

Eight‐week‐old male Sprague Dawley rats (SLC) weighing 200–260 g were maintained on a 12‐hr light/dark schedule and given ad libitum access to food and water. All experiments were performed in accordance with protocols approved by Tokoha University Animal Experiment Committee, based on the NIH Guidelines for the Case and Use of Laboratory Animals (Herling, Maas, Seeger, 1997). Rats were subjected to PIT according to procedures described previously with modifications (Umemura, Wada, Uematsu, & Nakashima, 1993). The body temperature of the animals was maintained at 37.5°C with a heating pad (003‐02B, Bio Research Center). Anesthesia was induced with 3.5% isoflurane in air. Subparietal craniotomy was performed using a dental drill under a microscope without damaging the lamina dura. The cranial window was centered 2 mm posterior and lateral to the Bregma point. The head of the optic fiber was placed on the window in the skull base. Immediately after rose bengal (20 mg/kg) was injected intravenously, the photo‐illumination of green light (wavelength, 540 nm) was achieved using a xenon lamp (model L‐4887, Hamamatsu Photonics) with heat absorbing and green filters. Irradiation was directed by an optic fiber 3 mm in diameter mounted on a micromanipulator. Photo‐illumination was performed for 10 min. The area of illumination included the motor (M1) regions. Sham‐operated rats, as a control for all aspects of the experimental surgery, underwent the same surgical procedure without the injection of rose bengal nor photo‐illumination.

### 2.2 Measurement of brain infarct

Brain infarct volumes were evaluated by 2,3,5‐triphenyltetrazolium chloride (TTC) staining. At two time points following the PIT operation (1 day and 4 weeks), rats were decapitated under deep anesthesia. After the brains were carefully removed, coronal brain slices (with a width of 2 mm) were cut with a brain slicer. The fresh brain slices were stained with 1% TTC at 37°C for 10 min. After images of TTC‐stained slices were acquired with a digital camera (D3200, Nikon), they were analyzed using ImageJ software (NIH). To minimize the error introduced by edema and liquefaction after infarction, a corrected percentage of the infarct volume was achieved using the following equation: corrected percentage of infarct volume = {(contralateral hemispheric volume − ipsilateral noninfarct volume)/contralateral hemispheric volume} × 100.

### 2.3 Experimental groups and exercise

First, all rats were subjected to a 5‐day adaptation to a motor‐driven treadmill (TMS, Melquest Ltd.) at a speed of 8 m/min. Next, rats were randomly assigned to one of the five experimental groups as follows: (a) low‐intensity treadmill exercise (PIT LowT, *n* = 19), (b) high‐intensity treadmill exercise (PIT HighT, *n* = 13), (c) voluntary exercise with wheel running (PIT V, *n* = 17), (d) nonexercise (PIT NonEx, *n* = 20), and (e) sham groups (*n* = 26). We also excluded rats that died before the end of each analysis and those in which an infarct core could not be detected after 4 weeks based on hematoxylin‐and‐eosin–stained sections.

One day after the PIT operation, exercise groups were subjected to different exercise paradigms for a total of 28 consecutive days. Rats in the PIT LowT and PIT HighT groups were forced to run on a treadmill for 30 min per day. Rats in the PIT LowT group were trained on the treadmill at a speed of 15 m/min, whereas rats in the PIT HighT group were subjected to a speed of 30 m/min. The running speed was gradually increased over 1 week after the PIT operation. For the PIT V group, each rat was housed in a cage in which they were freely able to access a running wheel apparatus (Kagawa Kikai) connected to a switch that counted each revolution of the wheel over 4 weeks. The average daily running distance travelled in the wheel apparatus was 953 ± 245 m/day in PIT V animals.

### 2.4 Determination of blood lactate levels

After blood samples (<5 μl) were collected from a small incision at the tip of the tail of the rats, blood lactate levels were determined from those samples using a lactate pro‐LT device (Arkray) in accordance with the manufacturer's instructions.

### 2.5 Neurological function

The neurological function of each animal was evaluated 24 hr after the photochemical reaction as previously described (Kawano, Fujishima, Ikeda, Kondo, & Umemura, 2000). The general posture of each animal was scored as follows: 0, normal; 1, the body was slightly shifted to right; and 2, their bodies were leaning markedly. In the dysfunction of paws, the fore paw, or hind paw, was pushed toward the body. The time to re‐extend the paw was scored as follows: 0, achieved within 1 s; 1, achieved within 5 s; and 2, not achieved within 5 s. In the postural reflex test, the animal was pushed in the contralateral direction and scored as follows: 0, normal; 1, slightly reduced resistance to lateral push; and 2, markedly reduced resistance to lateral push and fell down on the contralateral side. In the forelimb motor function, animals were held gently by the tail, and with their forelimb on the table, and their hind limbs suspended about 5 cm above the table. It was scored as follows: 0, animals normally walked; 1, animals usually walked ahead but not able to walk toward the left; and 2, animals walked toward the right and was not able to walk ahead. Each score was summed and represented as a neurological score (0–10).

### 2.6 Beam walking test

Motor coordination and recovery were evaluated to test hind limb abilities using a beam walking test. While rats traversed the surface of an elevated wooden beam (2.4 cm wide; 150 cm long) at a height of 30 cm above the ground, the performance was rated on a scale of 0–5 based on a modified scale criteria as follows: score 0, the rat was unable to traverse the beam; score 1–4, the rat traversed the beam with a distinct frequency (*F*) of foot slip (1: *F* ≥ 90%; 2: 70% ≤ *F* < 90%; 3: 50% ≤ *F* < 70%; 4: 20% ≤ *F* < 50%); and score 5, the rat traversed the beam with no or less than 20% slips. The mean of five trials at each time point was calculated.

### 2.7 Rotarod test

Motor recovery was also evaluated by measuring the latency with which the animal fell off an accelerating rotarod (LE8300; Panlab S. L; gradual acceleration 4–40 rpm per 5 min) once per week. The mean of five trials at each time point was calculated. We also performed this test on naïve rats (*n* = 3).

### 2.8 BrdU labeling

Animals were injected intraperitoneally daily with a single dose of the S‐phase marker 5‐bromo‐2′‐deoxyuridine (BrdU; 50 mg/kg body weight; Sigma‐Aldrich) for 7 days after the PIT operation. For each section, cells that showed incorporated BrdU into the DNA were detected with immunohistochemistry using a fluorescein‐conjugated secondary antibody, described as follows (Kumada, Lakshmana, & Komuro, 2006).

### 2.9 Immunohistochemistry

Rats were anesthetized with halothane and perfused transcardially with saline, which was followed by a freshly prepared solution of 4% paraformaldehyde (PFA, Sigma‐Aldrich) in 0.1 M phosphate buffer (PB), pH 7.4. The brains were rapidly removed, cut into approximately 2‐mm‐thick coronal slabs, and postfixed overnight in 4% PFA/0.1 M PB at 4°C. Coronal slabs were embedded in paraffin and cut serially at 4 µm with a microtome. Paraffin was removed from the sections, and specimens were heated in a microwave for 10 min in pH 6.0 sodium citrate buffer (REAL Target Retrieval Solution, Dako) to retrieve tissue antigens. For DNA denaturation, after gently cooling to 24–26°C, sections were incubated for 25 min with 5N HCl at 24–26°C and then incubated for 15 min with 0.1 M sodium tetraborate at 24–26°C.

Double immunofluorescent staining was performed as described previously with some modifications (Wang et al., 2014). The sections were incubated for 30 min with a rat polyclonal anti‐BrdU antibody (1:100; 5‐bromo‐2′‐deoxyuridine Labeling and Detection Kit II, Roche) at 37°C and then incubated for 30 min with Alexa Fluor 546‐conjugated goat anti‐mouse IgG secondary antibody (1:500; Thermo Fisher Scientific) at 37°C. Next, sections were incubated overnight with rabbit polyclonal anti‐Iba1 (1:500; Wako), rabbit polyclonal anti‐Sox2 (1:1,000; MBL), or rabbit polyclonal anti‐NeuN (1:500; Abcam) at 4°C. This was followed by incubation for 1 hr with Alexa Fluor 488‐conjugated goat anti‐rabbit IgG secondary antibody (1:500; Thermo Fisher Scientific) at 24 –26°C. After washing, the sections were mounted in Prolong Gold antifade (Thermo Fisher Scientific) and were observed using a fluorescence microscope (BX51; Olympus) equipped with a sCMOS camera (Andor Technology) or an all in one fluorescence microscope (Keyence BZ9000). Because certain images were obtained from a system with inverted microscopy (Figures 3a, 4a, 5a and 6a), these images were mirror‐inverted in software (Adobe Photoshop) to adjust the left–right orientation.

### 2.10 Cell counting

To quantify the number of cells in the ipsilateral cortex, we utilized combined tiled images (6 × 10^6^ μm^2^) after each image was acquired by microscopy with ×20 or ×10 objective lenses. After trimming out the region of the stroke core, the number of cells was automatically counted using ImageJ software for BrdU+/Iba1+, BrdU+/Sox2+, and BrdU+/NeuN+ double immunofluorescent signals. Each cell number represents the density in a fixed area (3 × 10^6^ mm^2^). Coronal sections located 1 mm posterior to the bregma were selected for immunofluorescent staining.

### 2.11 Statistical analysis

All values represent the mean ± standard error of the mean, except where indicated. One‐way analysis of variance (ANOVA) was used for multiple comparisons among groups. To test the effect of exercise on each behavioral test, we used the repeated measures analysis of variance (rANOVA). Results of beam walking scores were analyzed using Friedman's and Kruskal–Wallis nonparametrical tests. Post hoc comparisons (Tukey's, Scheffé's, or Steel's test) were carried out only when the primary measure showed statistical significance. The results were considered significantly different at *p* < .05.

## 3 RESULTS

### 3.1 TTC analysis of a rat model of focal cerebral stroke induced by PIT

**Figure 1:**
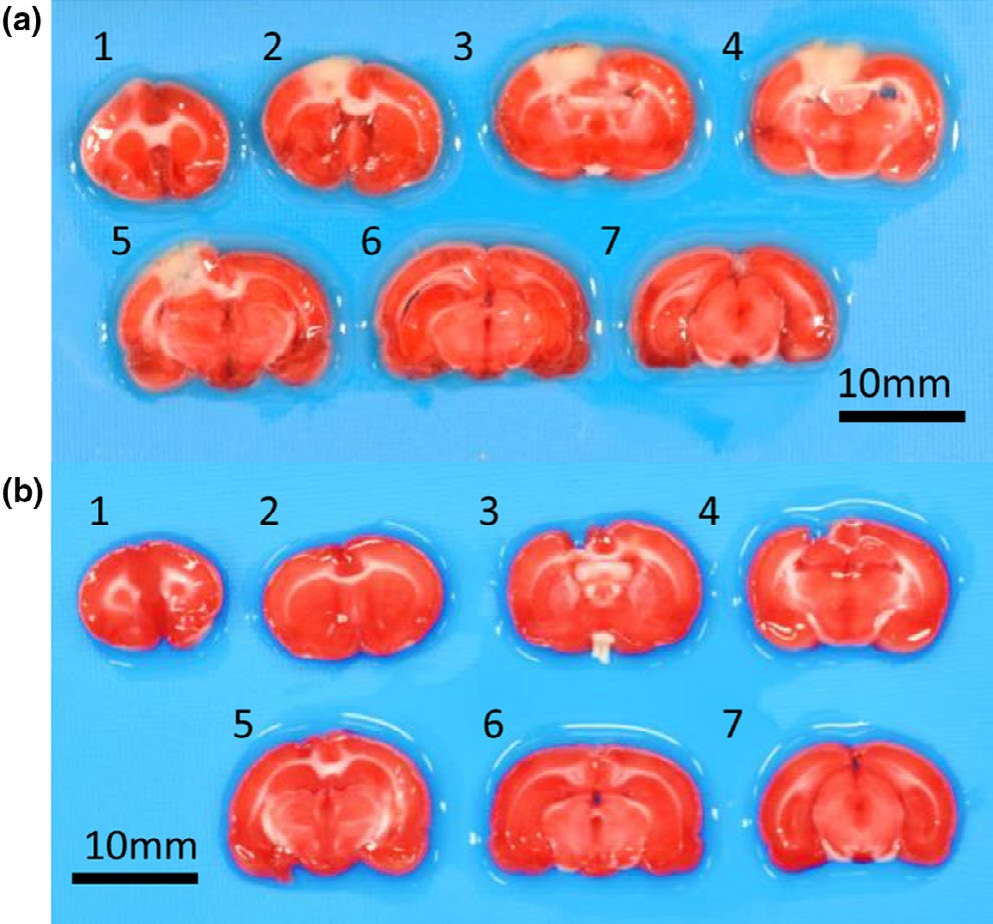
Representative images of 2,3,5‐triphenyltetrazolium chloride (TTC)‐stained coronal brain slices after 1 day (a) and 4 weeks (b) of photochemically induced thrombosis (PIT). Scale bar = 10 mm

To understand the mechanisms underlying the exercise‐mediated recovery of motor functions and neuronal plasticity in local neuronal circuits, we developed a rat model of focal cerebral infarction in the motor area using PIT. One day after the PIT operation, focal brain injury was observed in the medial parietal region of the left cortical hemisphere as shown in Figure 1a. Further, TTC staining of brain sections revealed that a narrow range of the medial parietal region, mainly confined to the limb region of the motor cortex, (Neafsey et al., 1986), was damaged, whereas other areas including subcortical regions appeared normal. The infarct area in the PIT groups was 8.4 ± 1.2% (*n* = 8) of the entire slice 1 day after operation (Figure 1a, Table S1). Four weeks after PIT operation, the infarct area had shrunken compared with 1 day after PIT (2.7 ± 3.3%, *n* = 5; Figure 1b, Table S1).

### 3.2 Effect of different types of exercise on rats and blood lactate levels

Altered lactate concentrations are known to be related to treadmill speed, and such thresholds are used to discriminate the intensity of activities such as aerobic and nonaerobic exercises (Abreu, Mendes, Leal‐Cardoso, & Ceccatto, 2016). To elucidate the exercise intensity of treadmill training, we measured blood lactate levels during activity. As shown in Figure S1, blood lactate levels gradually increased as the speed of running increased. We characterized the different intensity exercise programs into three groups—0 m/min (NonEx), 15 m/min (PIT LowT), and 30 m/min (PIT HighT)—with blood lactate levels at 1.8 ± 0.4, 2.4 ± 0.7, and 3.6 ± 0.7 mmol/L, respectively. We also measured body weight to elucidate the effects of exercise intensity on rats. Body weight gains in all exercise groups after focal cerebral infarction did not significantly change at both time points (1 day and 4 weeks after PIT operation) compared with those in the sham group.

### 3.3 Effect of different types of exercise on motor recovery

**Figure 2:**
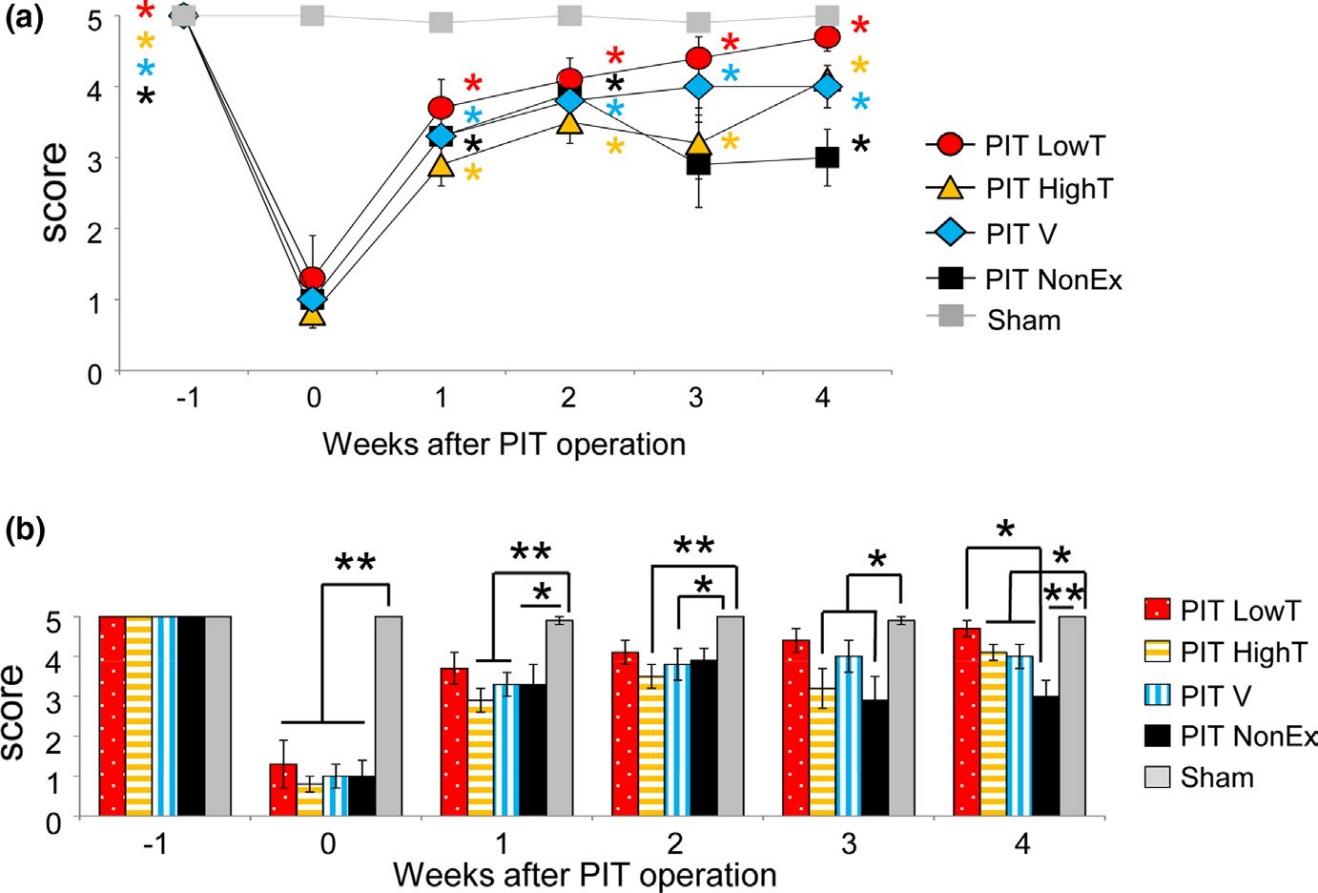
Motor recovery in photochemically induced thrombosis (PIT)‐operated rats subjected to different exercises based on the beam walking test. (a) Time course of beam walking scores in each group. Although the scores in different exercise groups were significantly increased at 3 and 4 weeks compared with 0 week after the operation (Friedman analysis, post hoc Steel's test compared with 0 week), the recovery process seemed to be different among the groups. (b) Comparison of beam walking scores among groups at different time points (Kruskal–Wallis analysis, post hoc Scheffé test). Note that the score in the LowT group was not significantly different from that in sham‐operated rats 1–4 weeks after the operation and was significantly different than that in the PIT NonEx group 4 weeks after the operation. In contrast, the score in the PIT NonEx group was significantly different than that in sham‐operated rats at 3 and 4 weeks after the operation. PIT HighT, high‐intensity treadmill exercise; PIT LowT, low‐intensity treadmill exercise; PIT NonEx, nonexercise; PIT V, voluntary exercise with wheel running

To elucidate the relationship between motor deficits and changes in local neural circuits, we first assessed the motor behavior of rats with PIT‐induced focal cerebral infarction. These animals displayed low scores in neurological testing (the median value was 1), suggesting that our model should exhibit mild motor deficits due to limited damage primarily to the motor cortex. We analyzed motor coordination and balance by performing a beam walking test with modified criteria as shown in Table 1. One day after PIT, neurological scores were significantly decreased in PIT‐operated rats compared with those in the sham group (Kruskal–Wallis analysis, post hoc Steel's test, compared with PIT, *p* < .05; Figure 2). Similarly, PIT‐operated rats exhibited significant decreases in the meantime on the accelerated rotarod compared with that in the sham group (Figure S2). These results suggested that our model rats exhibited motor deficits with respect to gait disturbances despite the induction of focal cortical infarction in the motor cortex.

**Table 1:** Beam balance test graded on a scale of 0–5 (minimum = 0; maximum = 5)

Score	Behavior
5 point	Traversed the beam with no or less than 20% slips
4 point	Traversed the beam with 20% or more and less than 50% slips
3 point	Traversed the beam with 50% or more and less than 70% slips
2 point	Traversed the beam with 70% or more and less than 90% slips
1 point	Traversed the beam with 90% or more slips
0 point	Unable to traverse the beam

Next, we determined what types of exercises contribute to motor recovery 4 weeks after PIT‐induced ischemic infarction. Starting from 1 day after the operation for 4 weeks, the exercise groups were forced to run on a treadmill with different intensities for 30 min every day or were subjected to voluntary wheel running. We evaluated rat motor behavior once per week based on a beam walking test. As shown in Figure 2a, neurological scores increased in the first 2 weeks in all groups of PIT‐operated rats. However, after 2 weeks postoperation, those in the nonexercise group declined slightly and subsequently maintained at a steady level (Figure 2a). In contrast, there were significant differences in the extent of functional recovery between the exercise groups (Figure 2b, Kruskal–Wallis analysis). Exercise resulted in further recovery even in the chronic phase. Among different exercise paradigms, low‐intensity treadmill exercise tended to facilitate motor recovery from an early stage (1 week) and significantly improved motor deficiencies compared with those in the nonexercise group. However, there was a slight delay in motor recovery in the high‐intensity treadmill training group. The efficacy of low‐intensity treadmill exercise was also confirmed by rotarod tests (Figure S2), although the effects seemed to be lesser.

### 3.4 Effect of exercises on *de novo* neurogenesis after ischemic infarction

**Figure 3:**
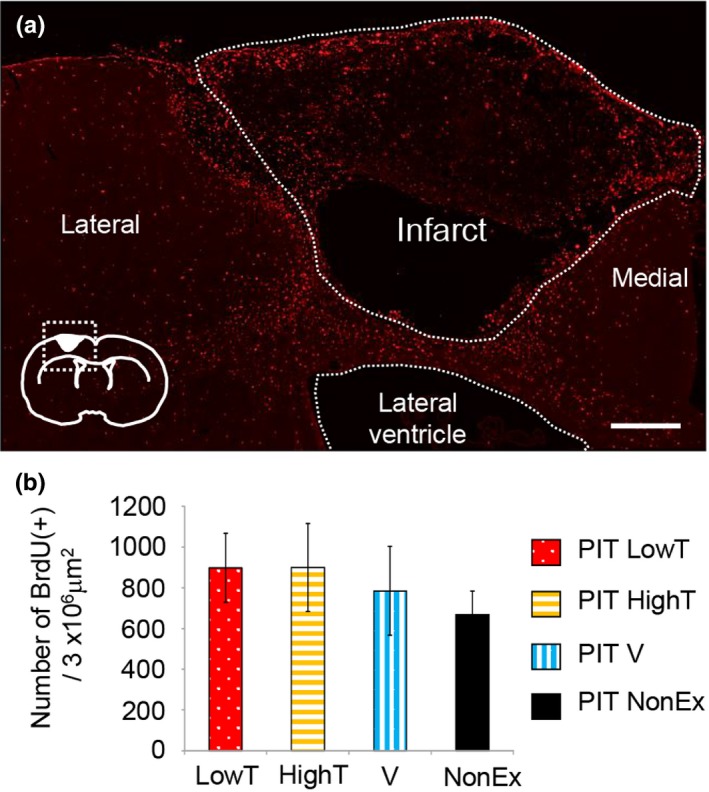
Effect of different exercises on distribution of BrdU‐positive cells in 4 weeks after PIT operation. (a) Distribution of BrdU immunoreactivity in the ipsilateral neocortex in the photochemically induced thrombosis (PIT) LowT (low‐intensity treadmill exercise group) rat group 4 weeks after the PIT operation. Inset, schema showing the approximate locations of the ROI (dotted square) and the stroke core. Scale bar = 300 μm. (b) Quantification of BrdU‐positive cells in the ipsilateral cortex (3 × 10^6^ μm^2^). Note that numbers of BrdU‐positive cells were not significantly different among the groups. In the regions around the ischemic core, especially at the lateral side, BrdU‐positive cells tended to be increased in the exercise group compared with those in the nonexercise group. The results are shown as mean ± *SEM* (one‐way factorial ANOVA followed by a Tukey's post hoc test)

To examine the effects of various types of exercises on the generation of new cells in the ipsilateral cortical regions of a rat model of focal motor cortex infarction, we quantified these cells by lineage analysis using BrdU. During the first week after PIT, we injected all groups intraperitoneally daily with BrdU (50 mg/kg body weight), which is incorporated only into proliferating cells such as neural stem cells. After sacrifice, we examined the distribution of newly formed BrdU‐labeled cells in ipsilateral cortical regions. In the sham group, BrdU‐positive immunoreactivity was only detected in the ventricular region of the examined SVZ. BrdU‐positive immunoreactivity was still observed in the peri‐infarct area 4 weeks after BrdU labeling experiments (Figure 3a). Furthermore, there was no significant difference in the number of BrdU‐positive cells in the ipsilateral cortex among all groups of infarct rats (ANOVA, post hoc Tukey's test）.

**Figure 4:**
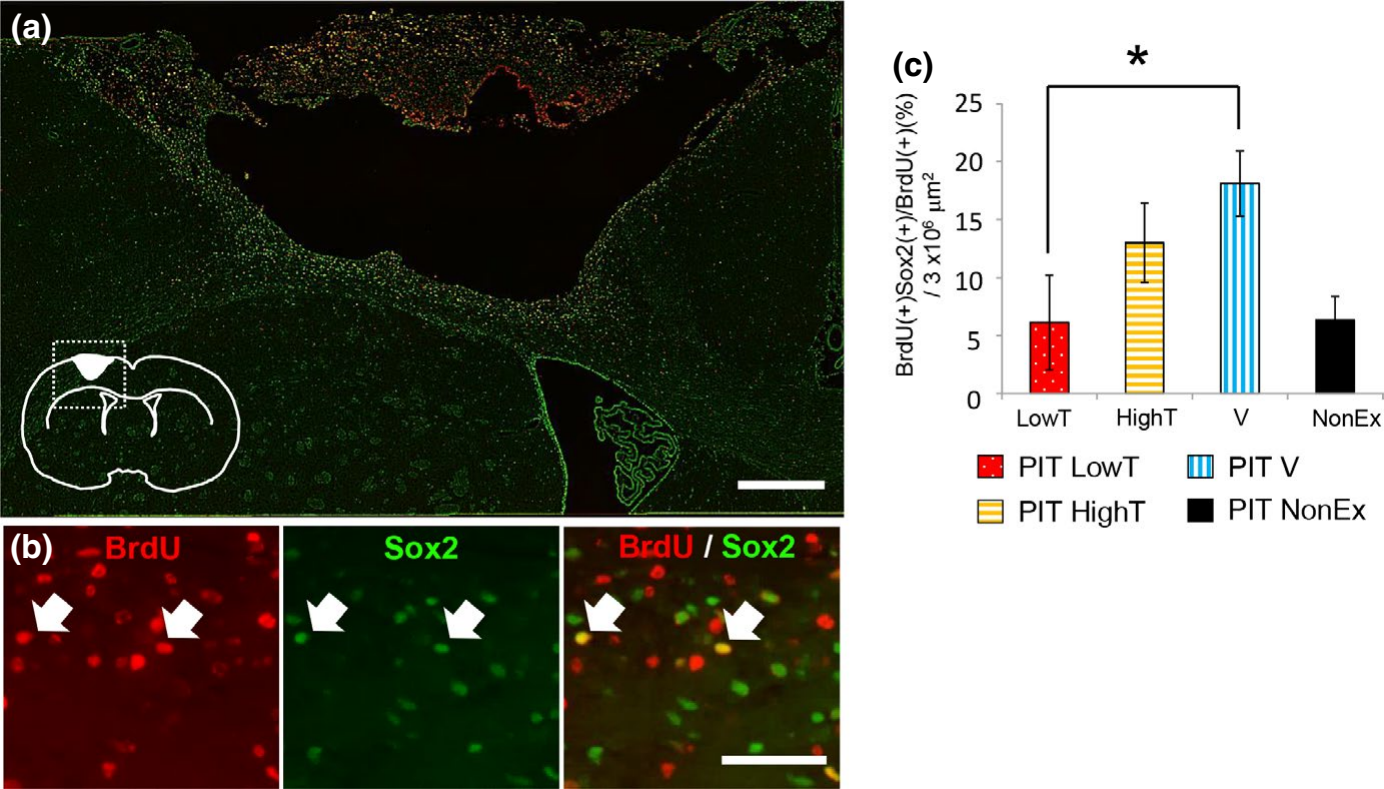
Effect of different exercises on number of BrdU(+), Sox2(+)‐double positive cells at 4 weeks after PIT operation. (a) Double immunostaining of BrdU (red) and Sox2 (green) in the ipsilateral neocortex of photochemically induced thrombosis (PIT) group rats subjected to different exercises at 4 weeks after the operation. Inset, schema showing the approximate locations of the ROI (dotted square) and the stroke core. Scale bar = 300 μm. (b) Higher magnification images. Arrows indicate colabeling signals for BrdU and Sox2. Scale bar = 50 μm. (c) Ratio of BrdU and Sox2‐double positive cells in the ipsilateral cortex. Note that BrdU and Sox2‐double positive cells were significantly increased in the PIT V (voluntary exercise with wheel running) groups compared with those in the PIT LowT (low‐intensity treadmill exercise group) group. The results are shown as the mean ± *SEM* (one‐way ANOVA followed by a Tukey's post hoc test, *p < .05)

Although different exercises did not affect the total number of newly generated cells in the ipsilateral cortex 4 weeks after PIT, there was still the possibility that they might induce the generation of distinct cell types. Therefore, we determined whether neuronal stem cells and their differentiation were influenced by different exercises. At first, we examined the number of Sox2 (a marker of neural stem cells) and BrdU‐double positive cells in the ipsilateral cortex of PIT‐operated rats subjected to different exercises. Both immunoreactivities were detected in a small area that should correspond to nuclei. Sox2 immunoreactivity was widely observed in both the SVZ and peri‐infarct region, and some of these populations colocalized with BrdU immunoreactivity (Figure 4). As shown in Figure 4, there was a significantly different number of Sox2/BrdU‐double positive cells among PIT‐induced rats with distinct types of exercises (ANOVA, post hoc Tukey's test, *p* < .05). Sox2/BrdU‐double positive cells were significantly increased in the PIT V group compared with that in the PIT LowT group, suggesting that BrdU‐incorporated neural stem cells might stop proliferating 4 weeks after infarction.

**Figure 5:**
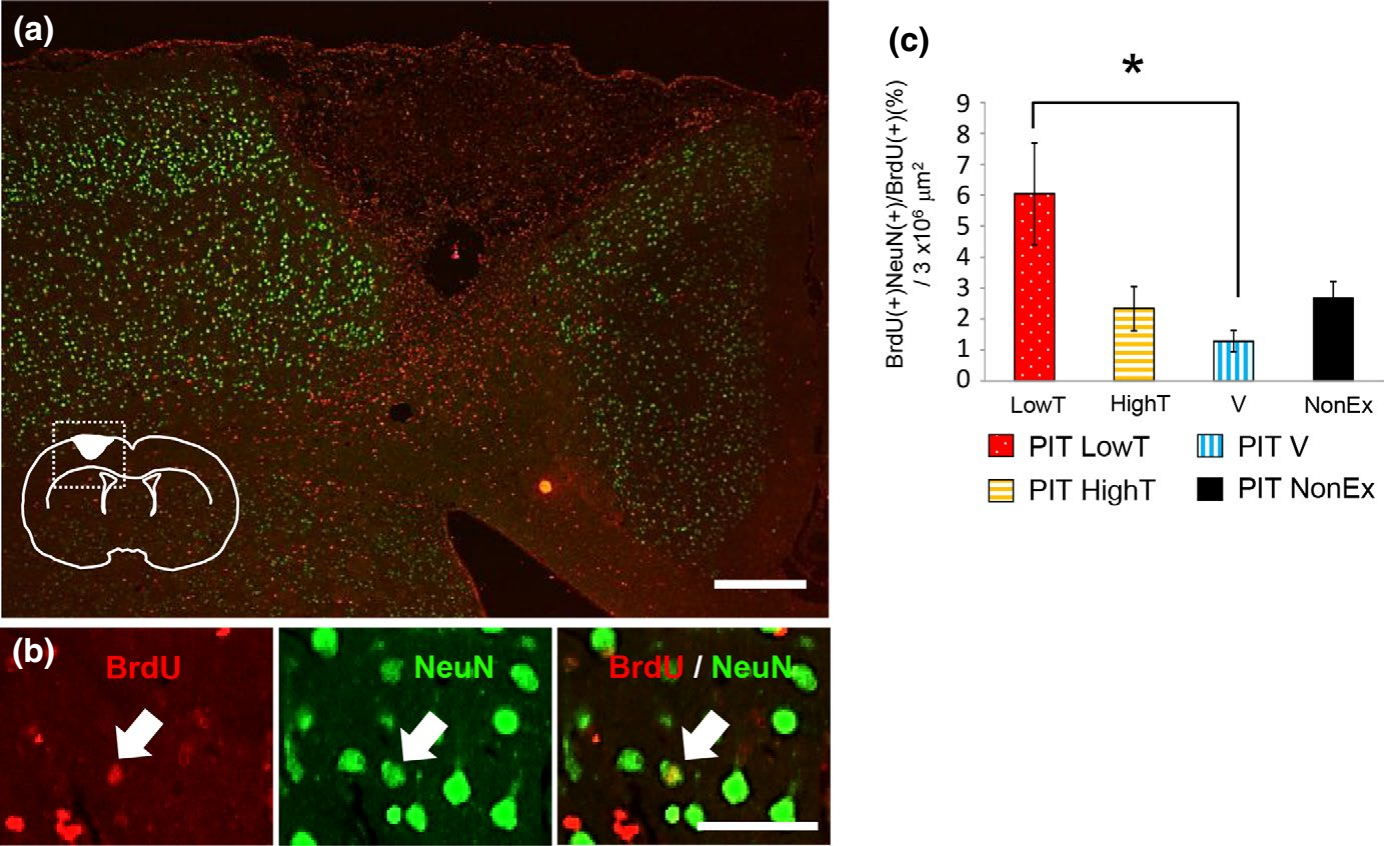
Effect of different exercises on number of BrdU(+), NeuN(+)‐double positive cells in 4 weeks after PIT operation. (a) Double immunostaining for BrdU (red) and NeuN (green) in the ipsilateral neocortex of photochemically induced thrombosis (PIT) group rats subjected to different exercises at 4 weeks after the operation. Inset, schema showing the approximate locations of the ROI (dotted square) and the stroke core. Scale bar = 300 μm. (b) Higher magnification. A small number of BrdU(+)/NeuN(+) cells were observed in the peri‐infarct region. Scale bar = 50 μm. (c) Ratio of BrdU and NeuN‐double positive cells in the ipsilateral cortex. Note that BrdU and NeuN‐double positive cells were significantly increased in the PIT LowT (low‐intensity treadmill exercise group) group compared with that in the PIT V (voluntary exercise with wheel running) group. The results are shown as the mean ± *SEM* (one‐way ANOVA followed by a Tukey's post hoc test, *p < .05)

Next, we determined whether different types of exercises affect *de novo* neurogenesis. Newly divided cells labeled with BrdU and NeuN (a marker for mature neurons) following daily BrdU administration between 1 and 7 days post‐PIT were detected by immunohistochemistry and BrdU and NeuN‐double positive cells were quantified in the ipsilateral cortex. NeuN immunoreactivity was observed even in the surrounding region close to the infarction. In this region, a small proportion of BrdU/NeuN‐labeled neurons was detected. The proportion of BrdU‐positive cells expressing NeuN was significantly different among PIT‐induced rats subjected to different exercises (ANOVA, post hoc Tukey's test, *p* < .05). Specifically, the PIT LowT group showed a significant increase in BrdU/NeuN‐double positive cells in the ipsilateral cortex compared with that in the PIT V group (Figure 5).

**Figure 6:**
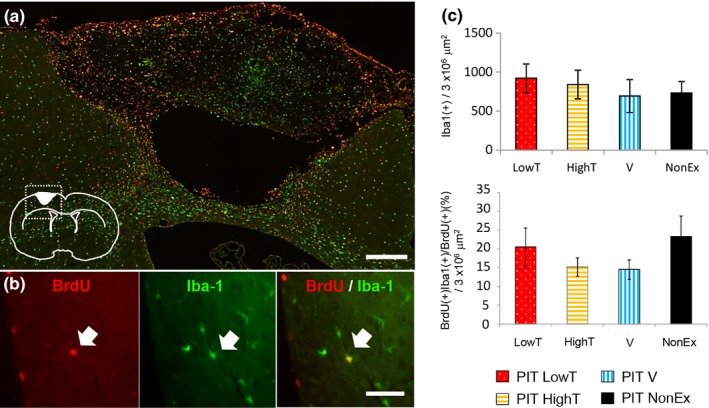
Effect of different exercises on number of BrdU(+), Iba‐1(+)‐double positive cells in 4 weeks after PIT operation. (a) Distribution of BrdU (red) and Iba‐1 (green) immunoreactivity in the ipsilateral neocortex of photochemically induced thrombosis (PIT) group rats subjected to different exercises at 4 weeks after the operation. Scale bar = 300 μm. (b) Higher magnification images. Arrows indicate colabeling signals of BrdU and Iba‐1. Scale bar = 50 μm. (c) Ratio of BrdU and Iba‐1‐double positive cells in the ipsilateral cortex. Note that the numbers of BrdU and Iba‐1‐double positive cells were not different among any exercise groups. The results are shown as the mean ± *SEM* (one‐way ANOVA, *p* = .5847)

There are reports that new cells are often damaged by neuronal inflammation and apoptotic cell death during the chronic phase in rodent models of cerebral stroke. We thus determined whether different types of exercises might distinctly affect neuronal inflammation. Microglial cells are well known to be recruited and affect both neuronal inflammation and neuroprotection after brain injury. Therefore, we examined the number of Iba1‐positive cells (a marker of microglial cells) in the ipsilateral cortex of PIT‐operated rats with different exercises. As shown in Figure 6, Iba‐1‐positive cells were not significantly different among all groups of infarct rats (ANOVA, post hoc Tukey's test), suggesting that microglia are not influenced by different exercises. Since we specifically focused on newly generated cells during the early stage of exercise (1 week postoperation), we also quantified the number of Iba‐1/BrdU‐double positive cells in the ipsilateral cortex of PIT‐operated rats after different exercise regimens. Iba‐1/BrdU‐double positive cells were still observed in the peri‐infarct region 4 weeks after PIT. However, exercise did not significantly affect the number of these cells.

## 4 DISCUSSION

In this study, we show that distinct exercises elicit motor recovery in a time course manner and affect the derivatives of newly generated cells 4 weeks after photochemically induced focal cerebral infarction in rats. Indeed, lower‐intensity treadmill (forced) exercise increased the number of BrdU/NeuN colabeled cells, but not the number of total BrdU‐positive cells and BrdU/Sox2 colabeled cells in the peri‐infarct region of the ipsilateral cortex. In contrast, high‐intensity treadmill and voluntary exercises had opposite effects.

Here, we tested three different exercises: (a) forced lower‐intensity and (b) higher‐intensity treadmill exercises, and (c) voluntary exercise with wheel running. This study provides evidence that lower‐intensity treadmill exercise has more beneficial effects on several aspects of recovery as compared to the other types. We discuss these as follows.

Firstly, beam walking analysis revealed that the rats subjected to lower‐intensity treadmill exercise showed augmented recovery from motor balance and coordination dysfunction compared to those with other exercises. Moreover, this analysis displayed distinct progressions in recovery from the different exercises, as well as in control conditions. Without exercise, the motor score was increased in the early phase of recovery (2 weeks after infarction), but was slightly reduced at 4 weeks, suggesting that improvements in motor recovery in the early and later stages might be regulated by different mechanisms. Motor compensation, which can be triggered by cerebral infarction and is generally considered to contribute to functional improvements, might affect recovery because it can have mixed effects on functional outcomes (Jones, 2017). Although motor recovery examined in this study was promoted by any exercise compared with no exercise, the extent was significantly different among exercises. The present study also suggested that lower‐intensity treadmill exercise results in earlier and sustainable improvement compared with other regimens. These results are partially consistent with a number of studies that compared the effects of exercises of different intensities on hippocampal memory function and some studies that explored recovery from ischemic stroke with respect to motor dysfunction (Inoue et al., 2015; Shih, Yang, & Wang, 2013; Shimada et al., 2013).

Secondly, our immunohistochemical studies showed that PIT‐mediated cerebral infarction markedly induced newly generated cells labeled with BrdU in the ipsilateral cortex. However, the total number of BrdU‐positive cells was not significantly changed among all groups of PIT‐operated rats. We further demonstrated that lower‐intensity treadmill exercise not only enhances motor recovery but also increases BrdU/NeuN‐double positive cells, which suggests that it might promote the differentiation or survival of newly generated neurons. These results are consistent with previous studies (Chen et al., 2019; Farioli‐Vecchioli et al., 2014; Jiang, Gu, Brannstrom, Rosqvist, & Wester, 2001). In contrast, higher‐intensity treadmill or wheel running exercises increased BrdU/Sox2‐double positive cells, but decreased BrdU/NeuN‐double positive cells, suggesting that the differentiation of newly generated neural stem cells could be perturbed by those exercises. To the best of our knowledge, the effect of exercise has been reported previously to either promote or inhibit adult neurogenesis based on other ischemic and hemorrhagic stroke models induced by MCAO or endothelin‐1 injection methods (Chen et al., 2019; Jiang et al., 2001; Leasure & Grider, 2010; Shimada et al., 2013). In the present study, we proposed that exercise could differentially affect the generation of new cells and differentiation. This discrepancy might be caused by different animal models. Here, we used a rat model of focal motor cortex infarction induced by the PIT method, which resulted in minor dysfunction. Accordingly, neurogenesis could be influenced by various conditions such as severity. For example, adult neurons have been found to be further generated from layer I of the neocortex in a nonsevere (mild) model of the ischemic brain (Ohira et al., 2010).

Immunohistochemical staining also showed many Iba‐1‐positive microglial cells and a population of BrdU/Iba‐1‐double positive cells in the ipsilateral cortex of PIT‐operated rats. This suggests that these cells could be involved in gliosis or inflammation (Burda & Sofroniew, 2014). However, the exercise conditions used in this study did not affect their populations.

In the current study, we determined the intensity of exercise based on blood lactate levels and specifically, the sub‐ and supralactate threshold, which is a known typical classification of training (Abreu et al., 2016). Body weights of PIT rats were not significantly different among all groups examined 4 weeks after exercise. In contrast, other studies utilized more intense exercises that caused a reduction in body weight (Inoue et al., 2015; Lezi, Burns, & Swerdlow, 2014; Shih et al., 2013). Thus, we speculate that our “higher‐intensity” exercise might be relatively low in intensity compared with other protocols.

Our data indicate that only a small number of neurons were increased upon exercise, and these data are consistent with the interpretation that exercise‐induced neurogenesis should not be sufficient to replace the neuronal circuit after injury (Arvidsson et al., 2002; Obernier, Tong, & Alvarez‐Buylla, 2014). However, our results could suggest that the generation of neural stem/progenitor cells and their differentiation might be modulated by rehabilitation‐mediated neural plasticity. There might be other possible reasons for the increased generation of new cells; however, these cells did not survive after 4 weeks, or the damaged cells would have taken up BrdU after injury (Kuan et al., 2004). However, this possibility is unlikely because TUNEL‐positive cells were not increased (data not shown). Presently, we were unable to clarify the aforementioned possibilities; therefore, further studies will be required due to the limitations of our study. Particularly, BrdU labeling experiments that label newly generated cells can only track the populations of such cells in different time windows. Furthermore, we were not able to detect faint BrdU signals after repeated proliferation. In summary, a better understanding of the detailed relationship among exercise conditions, motor recovery, and neurogenesis could uncover clues to better elucidate the role of physical rehabilitation in regenerative medicine.

## CONFLICT OF INTEREST

The authors declare that the research was conducted in the absence of any commercial or financial relationships that could be construed as a potential conflict of interest.

## Data Availability

The data that support the findings of this study are available from the corresponding author upon reasonable request.
