# Supplementary figures and images for: Different exercises can modulate the differentiation/maturation of neural stem/progenitor cells after photochemically induced focal cerebral infarction
Source: Brain Behav. 2020 Jan 27;10(3):e01535. doi: 10.1002/brb3.1535 (PMC7066356; doi:10.1002/brb3.1535)

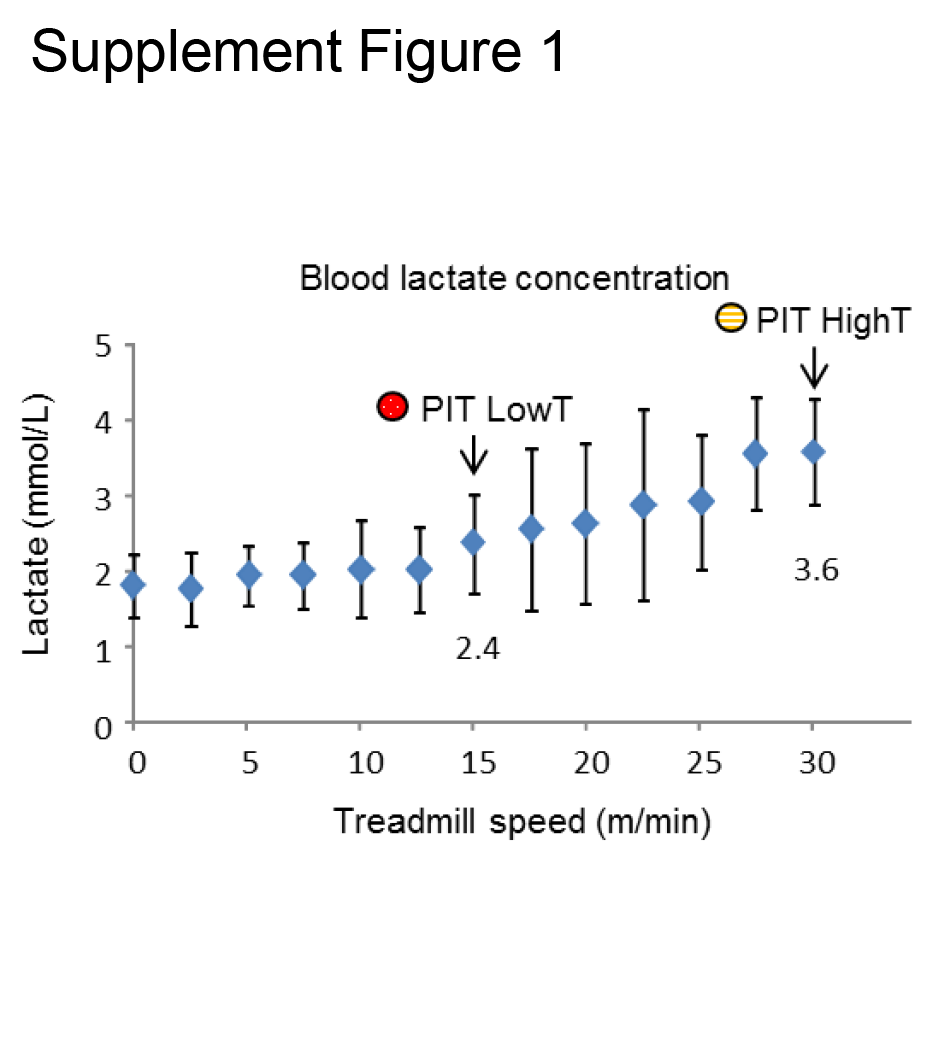

Supplement: Supplementary file 1 [file BRB3-10-e01535-s001.tif]

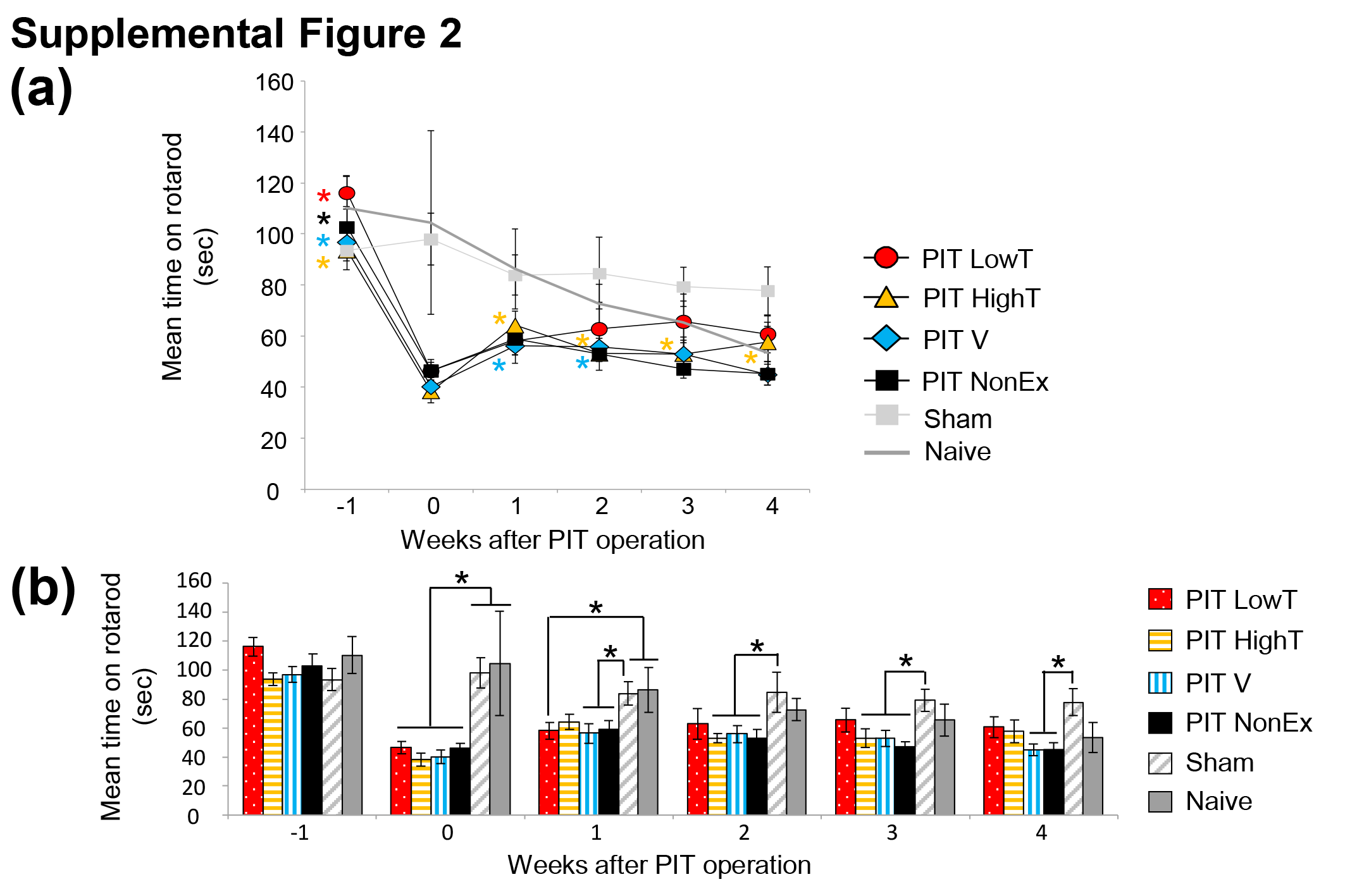

Supplement: Supplementary file 2 [file BRB3-10-e01535-s002.tif]
