# Supplementary material for: Different exercises can modulate the differentiation/maturation of neural stem/progenitor cells after photochemically induced focal cerebral infarction
Source: Brain Behav. 2020 Jan 27;10(3):e01535. doi: 10.1002/brb3.1535 (PMC7066356; doi:10.1002/brb3.1535)
Supplement: Supplementary file 3 [file BRB3-10-e01535-s003.docx]

**Supplemental Table 1 Infarct area (%)**

|  | Slice No. | 1 | 2 | 3 | 4 | 5 | 6 | 7 |
| --- | --- | --- | --- | --- | --- | --- | --- | --- |
| 1 day  After PIT  (n=8) | Average | 0.5 | 7.4 | 8.4 | 6.8 | 4.8 | 0.4 | 0.0 |
|  | SD | 1.0 | 2.2 | 1.2 | 3.1 | 4.1 | 1.0 | 0.0 |
| 4 weeks  After PIT  (n=5) | Average | 0.0 | 0.0 | 2.7 | 4.5 | 3.6 | 1.8 | 0.0 |
|  | SD | 0.0 | 0.0 | 3.3 | 1.3 | 1.8 | 1.9 | 0.0 |

**Supplemental Table 1** Quantification of the infarct area. Seven serial brain slices (2-mm thickness) were obtained from each animal and stained with TTC. A corrected percentage of the infarct area was achieved as described in *Materials and Methods*. Slice No. indicates the different positions of slices (1: anterior, 7: posterior)

**Supplemental Figure 1** Relationship between the speed of treadmill exercise and blood lactate levels.

**Supplemental Figure 2** (a)Time course of meantime on rotarod in each group (one-way repeated ANOVA with post hoc Dunnett’s test compared with 0 week). (b) Differences in meantime on rotarod among the groups subjected to different exercises at 1 to 4 weeks after the operation (one-way ANOVA with a post hoc Tukey’s test). Data are shown as the mean ± SEM (*P < 0.05).
